# Palliative care interventions and outcome in patients with glioblastoma – a retrospective, single-center study

**DOI:** 10.1186/s12904-026-01987-4

**Published:** 2026-01-16

**Authors:** Lisa-Marie Lind, Anna Fischl, Elisabeth Goettl, Wolfgang Herr, Ulrich Kaiser, Oliver Koelbl, Ralf Linker, Julia Maurer, Markus J. Riemenschneider, Nils-Ole Schmidt, Martin Proescholdt, Tobias Pukrop, Peter Hau, Michael Rechenmacher, Elisabeth Bumes

**Affiliations:** 1 Department of Neurology – NeuroOncology and Wilhelm Sander-NeuroOncology Unit, Regensburg University Hospital, Regensburg, Germany; 2 University Cancer Center Regensburg, Regensburg University Hospital, Regensburg, Germany; 3 Bavarian Cancer Research Center (BZKF), Regensburg, Germany; 4 Department of Internal Medicine III – Hematology and Oncology, Regensburg University Hospital, Regensburg, Germany; 5 Adiuvantes SAPV, Landshut, Germany; 6 Department of Radiotherapy and Radiooncology, Regensburg University Hospital, Regensburg, Germany; 7 Department of Neuropathology, Regensburg University Hospital, Regensburg, Germany; 8 Department of Neurosurgery, Regensburg University Hospital, Regensburg, Germany; 9 Center for Palliative Medicine, Regensburg University Hospital, Regensburg, Germany

**Keywords:** Brain tumor, Glioblastoma, Palliative care, Primary palliative care, Specialized palliative care, Neuropalliative care, Overall survival

## Abstract

**Background:**

Patients with glioblastoma (GB) not only suffer from a life-threatening oncological disease but also present with severe neurological symptoms and high psychosocial distress. The unfavorable prognosis and the early decline in neurological functions and activities of daily living, such as mobility, lead to a significant deterioration in quality of life aspects. The need for palliative care (PC) therefore arises at an early stage and increases as the disease progresses but is often inadequately assessed and treated.

**Methods:**

In this single-center, retrospective study, we investigated prognostic factors, survival outcomes and neuro-oncologically focused primary palliative care (nPPC) as well as specialized palliative care (SPC) interventions. Pearson’s Chi-square test and an univariable and multivariable binary logistic regression analysis were used to test the independence between categorical variables and the correlation between SPC and tumor-specific therapy prior to death. The Kaplan-Meier method and a multivariable Cox regression analysis were performed to estimate the impact of SPC on survival.

**Results:**

A cohort of 274 patients with GB was investigated, of whom 251 (91.6%) received nPPC and 210 (76.6%) SPC. Patients with SPC (*p* < 0.001; OR: 0.302; 95% CI: 0.157–0.584) and patients with methylation of the *MGMT* promoter region (*p* = 0.005; OR: 0.375; 95% CI: 0.190–0.739) were less likely to receive a tumor-specific therapy in the 30 days prior to death. Median overall survival was 16.9 months (95% CI: 14.5–19.3 months) for patients with SPC (*n* = 210) vs. 12.9 months (95% CI: 10.8–15.1 months) for patients without (*n* = 64) (*p* = 0.100; not significant). The Cox proportional hazards model demonstrated that SPC significantly correlates with longer overall survival (*p* = 0.017; HR: 0.707; 95% CI: 0.532–0.939).

**Conclusion:**

This study revealed a broad availability of PC interventions for patients with GB. After adjustment of known prognostic factors, an association between SPC supply and prolonged OS was observed. Utmost efforts should be made to incorporate PC into the care of every patient within a standardized framework. Data on PC in patients with GB is still rare; therefore, further research should be made to improve PC in this highly burdened patient group.

**Supplementary Information:**

The online version contains supplementary material available at 10.1186/s12904-026-01987-4.

## Introduction

Glioblastoma (GB), isocitrate dehydrogenase wild type (IDHwt) (CNS WHO grade 4) [1], is the most common primary brain tumor diagnosed in adults [2]. The median overall survival (mOS) of all patients with GB remains unfavorable at 15–48 months according to meaningful clinical studies (depending on *O-6-methylguanine-DNA methyltransferase* (MGMT) promoter methylation status, treatment regimen, and WHO classification applied) [3–5]. Real-world data from the most recent CBTRUS report (2018–2022) reported a mOS of 8 months in patients with GB in the United States, with a one-year survival rate of only 48.7% [2]. Among therapy-independent prognostic factors, sex, age at first diagnosis, extent of resection, Karnofsky Performance Status Scale (KPS) at first diagnosis [4] and *MGMT* promoter methylation status are important prognostic factors [3].

Patients with GB not only suffer from a life-threatening oncological disease, but also from a severe neurological symptom burden including intracranial pressure, epileptic seizures, focal neurological symptoms such as hemiparesis and aphasia, neurocognitive dysfunction and personality changes as well as side effects of tumor therapies [6–10]. In addition to symptoms, patients feel distressed by limitations of their mobility, ability to work, social network and enjoyment of life [6, 11]. This entails a high psychosocial and emotional burden on patients and their caregivers, leading to reduced health-related quality of life (HR-QoL) [12–14].

Owing to the palliative character of GB, there is a high demand for palliative care (PC) throughout the course of the disease [15]. Here, primary palliative care (PPC) as well as specialized palliative care (SPC) are crucial [16]. PPC is provided by any medical practitioner and remains an integral part of standard care for critically ill patients. It covers basic symptom management (e.g. pain, depression) and initiates first conversations about prognosis and end of life. In specialized neuro-oncological care, this is frequently undertaken by neuro-oncologists, so we refer to this as neuro-oncologically focused primary palliative care (nPPC).

In contrast, SPC is carried out by palliative care specialists. SPC intervenes when symptoms are complex and the course of disease requires intensified psychosocial management [16–18]. SPC occurs in various inpatient and outpatient settings that all ensure integrative and interdisciplinary PC [19–21]. Inpatient palliative consultations are not equally accessible to all inpatients due to regional and structural differences [20]. Specialized palliative outpatient care (SPOC) tries to prevent hospitalization, enabling patients to live and die in their familiar environment [21].

The need for PC is often inadequately recognized, as assessment of symptoms is time-consuming and warrants awareness of the problem. As part of neuro-oncological certification systems, a screening for PC needs is often included [22]. Also based on these innovations, PC in neuro-oncology has gained more attention in recent years [18]. However, the evidence on PC - in particular on early PC - for patients with GB is still limited [23] and despite the overall intense research on glioma, topics on end-of-life care, HR-QoL, and psychosocial aspects are still underfocused [24]. The EANO guideline on palliative care of 2017 was the first that includes standards on symptom management, needs of patients and their caregivers and end-of-life care in neuro-oncology [25].

Still, studies are heterogenous and mostly retrospective in their design and show that the prevalence of SPC in patients with glioma differs substantially, with rates between 3% and 40%, depending on the tumor entities included and the treatment situation [26–29]. In most studies, SPC is integrated late in the course of the disease. In one study from 2013, the median time from first diagnosis to SPC referral was 111 days, whereas the median time from SPC referral to death was only 33 days [9]. More recent data also demonstrate that patients with high grade glioma are only referred to SPC in the end-of-life phase [27]. Overall, the number of patients who receive early PC remains small [30]. A recent prospective clinical study investigated the influence of early integration of PC (< 4 weeks after first diagnosis/recurrent diagnosis) on QoL of patients with GB and caregiver burden in a two-arm, randomized setting [31]. An analysis adjusted for time of death demonstrated an improvement in QoL of patients in the intervention group with early integration of PC. Data on survival outcomes for patients with GB depending on receiving PC are heterogeneous. The above-mentioned prospective study indicated a reduced OS for the intervention group with early integration of PC compared to the control group [31]. A retrospective analysis by Pando et al. including 85.380 patients with GB diagnosed between 2004 and 2017 observed a shorter two-year-survival in patients with PC compared to patients without PC, although it should be noted that PC was only observed in 3.28% of the study population [29]. Wu et al. retrospectively investigated data from 1997 to 2016 and found that the median survival time for GB patients with early PC integration was shorter than for patients without PC, while longer median survival times were observed in patients with late PC integration [30]. However, a retrospective analysis by Crooms et al. indicates that there may be a survival advantage for patients with high-grade gliomas who had early PC consultation compared to PC consultation at the end of life [27]. Furthermore, a systematic review by Koekkoek et al. concludes that the (early) integration of PC is essential to adequately address the needs of patients and caregivers, but the data available is still considered sparse [32].

Overall, data on PC in neuro-oncology are still sparse and heterogenous. Hardly any study examines both PPC and the various forms of SPC. Therefore, we investigated a range of PC interventions in patients with GB, which were related to prognostic factors as well as outcomes, providing a basis for a rational approach to PC in patients with GB.

## Materials and methods

### Study population

**Fig. 1:**
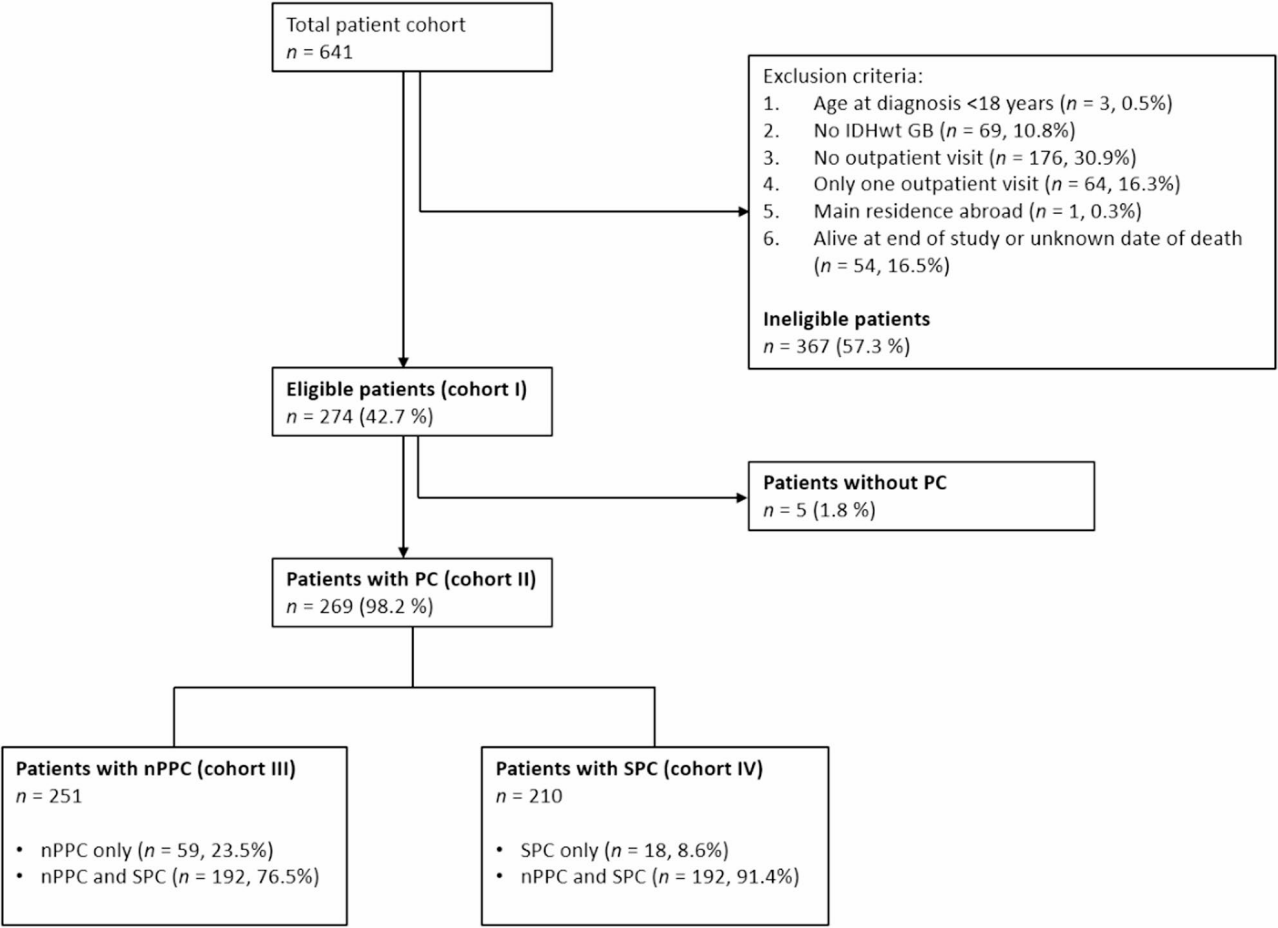
Patient cohorts regarding inclusion and exclusion criteria. Abbreviations: IDHwt, Isocitrate dehydrogenase wild type; PC, palliative care; nPPC, neuro-oncologically focused primary palliative care; SPC, specialized palliative care

Patients with GB, IDHwt (CNS WHO Grade 4) [1] who were registered in the local tumor registry between January 2014 and February 2024 and filed in our hospital data management system were included in this retrospective single-center study. Main inclusion criteria were an age at diagnosis of 18 or above, a neuropathological diagnosis of GB, according to the WHO classification of 2021, at least two outpatient visits after diagnosis, main residence in Germany and death before end of study (Fig. 1). Patients who were still alive at the time of the last data assessment were excluded for statistical reasons, as the data on PC interventions is incomplete and censoring can lead to an overestimation or underestimation of survival times.

Figure 1 lists the exclusion criteria in more detail according to the number of patients and reasons for exclusion. Cohort I comprises all eligible patients, which are divided into Cohort II (patients with PC) and patients without PC. Cohort III includes all patients who received nPPC, while Cohort IV represents patients with SPC (detailed information is provided in Fig. 1).

### Sociodemographic, clinical and treatment factors

Important demographic factors included date of birth, sex, age at first diagnosis, place of main residence and place of death. According to the WHO classification of 2021 [1], the histological or molecular diagnosis of GB was assessed. Patients who were initially diagnosed before 2021 underwent neuropathological reclassification according to the criteria of the 2021 WHO classification. Sex, age at first diagnosis, extent of resection, KPS at first diagnosis [33] and *MGMT* promoter methylation status were recorded as relevant prognostic factors. KPS is an established tool for assessing functional status in neuro-oncology and is an important aspect of prognosis assessment and treatment decisions [34]. Clinical and treatment-related aspects comprised primary therapy as well as date and type of any relapse/progression therapy, and the KPS at the respective stage of therapy. The last documented KPS refers to the most recent tumor progression in each patient. All abovementioned parameters were drawn from the electronic medical records.

### Primary palliative care

In the context of this study, nPPC refers exclusively to the PPC that has been provided by neuro-oncologists at the Regensburg Brain Tumor Center. To qualify as nPPC, symptoms were not treatment-related side effects and were not based on the pre-morbid medical history of the patient. The start date of nPPC, initial symptoms and medical interventions were assessed and documented from electronic patient records. The symptoms were assigned to categories in line with the SPOC prescription, which were divided into subcategories in consultation with a panel of experts consisting of neuro-oncologists and PC specialists (Supplementary Table 1). Validity checks were carried out repeatedly to ensure good inter-rater reliability. For this purpose, data was assessed, and symptoms were assigned to categories/subcategories independently by two researchers (LML and EB). Possible discrepancies were discussed and led to modification.

### Specialized palliative care

In view of SPC, the start date of SPC and all types of SPC interventions were further evaluated in detail. For inpatient palliative consultation, the start date, number of contacts and indications were drawn, and the reasons for consultation were assessed as symptoms of complex palliative care, consultation at first diagnosis, therapy goal setting, care coordination and other indications (Supplementary Table 2).

In evaluating the outpatient sector, the respective SPOC teams were individually queried. Patients were allocated to at least one SPOC team according to the postal codes of their main residence. An indication for SPOC is typically based on severe pain and/or neurological/psychiatric/psychological, cardiac/respiratory or gastrointestinal symptoms, ulcerating/exulcerating wounds/tumors, urogenital symptoms and some other symptoms (Supplementary Table 3) [35]. The survey also included standardized questions on start, duration and indication of SPOC, and number of in-person and phone contacts with SPOC. We further queried data on stays in a palliative care unit (PCU), hospice care and place of death. For patients who were not cared for by SPOC, we contacted their primary care physician for further information.

### Study endpoints

The endpoints of our study include the receipt of tumor-specific therapy within the last 30 days prior to death, the OS and progression-free survival (PFS). Tumor-specific therapy contains interventions such as surgery, system therapy and radiotherapy. Supportive therapy such as antiseizure medication, corticosteroids and bevacizumab as therapy for cerebral edema was excluded. The PFS was defined as the time from diagnosis up to first relapse/progression or death.

### Statistics

Pseudonymized data were assembled and recorded in IBM SPSS (Chicago, IL, USA) Statistics (Version 29). Continuous data was displayed as means, medians, standard deviations, minima, and maxima. A Student’s t-test was applied to compare the mean values in the case of a normal distribution of continuous variables. Categorical variables were characterized as absolute frequencies and relative percentages. The Pearson’s chi-square test was performed to test the independence between categorical variables. The Fisher’s exact test was applied if the sample size was too small. The level of significance was set at *p* < 0.05.

The correlation between SPC and tumor-specific therapy within the last 30 days before death was investigated with an univariable and multivariable binary logistic regression analysis. Possible prognostic confounders were assessed. A cut-off p-value of *p* < 0.2 in the univariable binary logistic regression analysis was defined as a selection criterion for possible confounders for the multivariable binary logistic regression analysis. The level of significance was set at *p* < 0.05 in the multivariable analysis.

The Kaplan-Meier method was used to estimate OS and PFS. Differences between groups were compared using the two-sided log-rank test with a level of significance at *p* < 0.05. A Cox proportional-hazards regression model was applied to assess the impact of SPC on OS and PFS. As possible confounders were included: sex, age at diagnosis, KPS at first diagnosis, *MGMT* promoter methylation and extent of resection. Parameters which were significant at the *p* < 0.2 level in the univariable cox regression analysis were included in the multivariable analysis. The level of significance was set at *p* < 0.05.

We defined SPC exposure as receipt of one or more SPC interventions after first diagnosis and therefore treated SPC as a time-fixed covariate (ever vs. never during the window). This reflects our interest in the association between receipt of SPC services and OS from diagnosis. Alternative approaches such as landmark analyses or time-dependent Cox models that treat SPC initiation as a time-varying treatment answer different causal contrasts (effects of initiating SPC at a particular time among survivors, or immediate hazard changes after SPC start) and are vulnerable here to selection bias or confounding by indication given that SPC is an ongoing, non-randomized service typically initiated in response to clinical deterioration.

### Ethical and regulatory framework

The study was approved by the Regensburg University Institutional Ethics Review Board (vote no. 24-3606-104). Owing to the retrospective character of this analysis, no informed consent was required in accordance with local regulations. The data protection concept of the Department of Neurology – NeuroOncology at Regensburg Brain Tumor Center, which operates within the framework of the European General Data Protection Regulation and relevant national legislation, was applied. Our study was conducted in accordance with the STROBE checklist for observational studies (Supplementary Table 4).

## Results

### Patient cohorts and characteristics

A data query at the Regional Cancer Center identified 641 patients who were diagnosed with GB between January 01, 2014, and February 28, 2024, of whom 274 (42.7%) met the pre-specified inclusion criteria (Fig. 1, cohort I). Data on nPPC were available in all patients. Amongst them, we identified 138 patients with SPOC (50.4%); data were acquired in 137 of them (99.3%). A data request was sent to the primary care physician for patients who could not be identified by a SPOC team in 136 cases (49.6%), and 121 (89.0%) primary care physicians fully completed the request.

In cohort I, 158 (57.7%) patients were male and 151 (55.1%) were older than 60 years at first diagnosis. The *MGMT* promoter was methylated in 117 patients (42.7%), and 183 (66.8%) patients presented with a KPS of 90 or above, 79 (28.8%) patients with a KPS of 70–80, and 12 (4.4%) with a KPS below 70 at first diagnosis. Two hundred and sixty-nine (98.2%) patients received either nPPC or SPC or both (cohort II); of these, 251 patients received nPPC (cohort III; including nPPC only, *n* = 59, and combined nPPC/SPC, *n* = 192) and 210 patients SPC (cohort IV; including SPC only, *n* = 18, and combined nPPC/SPC, *n* = 192) (Table 1, Supplementary Tables 1 and 2). Age at first diagnosis and sex did not differ notably from the overall cohort (Supplementary Tables 5 and 6). Details on the course of therapy can be obtained from the authors upon reasonable request.

**Table 1:** Demographic and clinical characteristics of cohort I (*n* = 274)

		Valid Number	Percent
Age at first diagnosis (years)	20.0–49.9	33	12.1%
	50.0–59.9	90	32.8%
	60.0–69.9	95	34.7%
	≥ 70	56	20.4%
Sex	Male	158	57.7%
	Female	116	42.3%
*MGMT* Methylation Status	No Methylation	157	57.3%
	Methylation	117	42.7%
KPS at first diagnosis	< 70	12	4.4%
	70–80	79	28.8%
	90–100	183	66.8%
Epilepsy	No	75	27.4%
	Yes	199	72.6%
Psycho-oncological care	No	153	55.8%
	Yes	121	44.2%
nPPC and SPC	Neither nor	5	1.8%
	Yes, both	192	70.1%
	Only nPPC	59	21.5%
	Only SPC	18	6.6%
nPPC	No	23	8.4%
	Yes	251	91.6%
SPC	No	64	23.4%
	Yes	210	76.6%
Place of death	At home	111	40.5%
	PCU	50	18.2%
	Hospice	31	11.3%
	Hospital	31	11.3%
	Nursing home	12	4.5%
	No data available	39	14.2%
	Total	274	100%

*Abbreviations: MGMT* O-6-methylguanine-DNA methyltransferase, *KPS* Karnofsky Performance Scale, *nPPC* Neuro-oncologically focused primary palliative care, *SPC* Specialized palliative care, *PCU* Palliative care unit

### Place of death

In cohort I, 111 (40.5%) patients deceased at home, whereas 50 (18.2%) patients deceased at a PCU, 31 (11.3%) at hospice, 12 (4.4%) patients in a nursing home and 31 (11.3%) in other medical departments of a hospital (Table 1). Details on cohort III and IV are shown in Supplementary Tables 5 and 6. In cohort IV, we further investigated whether SPC correlates with the place of death. Patients who deceased out of hospital received significantly more often SPC (89.0% vs. 75.3%; *p* = 0.006) (Table 2).

**Table 2:** Place of death in relation to SPC

		Place of death in hospital						
		No		Yes		Total		X^2^
		*n*	(%)	*n*	(%)	*n*	(%)	*p*
SPC	No	17	11.0%	20	24.7%	37	15.7%	0.006
	Yes	137	89.0%	61	75.3%	198	84.3%	
	Total	154	100.0%	81	100.0%	235	100.0%	

*Abbreviation: n* Valid number, *X2* Pearson’s Chi-square test

### Neuro-oncologically focused primary palliative care (nPPC)

#### Characteristics of nPPC interventions

At the beginning of nPPC, 177 (70.5%) patients in cohort III were without relapse/progression, and 120 (47.8%) presented with a KPS of 90 or above (Supplementary Table 5). The mean time from first diagnosis to start of nPPC was 22.5 weeks; 21 (8.4%) patients received nPPC already before the initial diagnosis, 51 (20.3%) within the first week of diagnosis and 59 (23.5%) within 1–10 weeks. Patients obtained their first nPPC intervention on average 13 months before death, with 109 (43.4%) patients with their first nPPC intervention more than 12 months before death (Supplementary Table 5).

#### Symptoms and indications of nPPC

The most common symptom that did lead to the first nPPC intervention was seizures in 55 patients (21.9%). Additional nPPC interventions were caused by other neurological symptoms in 122 patients (48.6%) and non-neurological symptoms in 74 patients (29.5%) (Supplementary Table 7). During the entire course of disease, the most common indications of nPPC interventions were neurological symptoms in 241 patients (96%), followed by psychiatric symptoms in 138 patients (55.0%) and pain in 63 patients (25.1%) (Supplementary Table 8).

### Specialized palliative care (SPC)

At the beginning of SPC, 66 (31.4%) patients presented with a KPS below 70 and a majority of 126 (60.0%) patients were in second or later relapse/progression (Supplementary Table 6). The mean time from first diagnosis to start of SPC was 72.4 weeks. Within 9–50 weeks of first diagnosis, 77 (36.7%) patients received their first SPC contact, whereas only 8 (3.8%) patients had their first contact within 8 weeks after diagnosis. The mean period from the start of SPC to death was 13.3 weeks, whereas 78 (37.1%) patients received their first SPC intervention within 3–10 weeks before death (Supplementary Table 6).

In cohort IV, 129 (61.4%) patients received inpatient SPC, of these 80 (38.2%) with inpatient palliative consultation and 82 (39.0%) on PCU (Supplementary Table 6). In the outpatient setting, 138 (65.7%) patients received long-time care from a SPOC, while 13 (6.2%) patients had a single contact with SPOC. A total of 31 (14.8%) patients were taken care of in the hospice (Supplementary Tables 6 and 9 to 15).

### SPC and tumor-specific treatment at the end of life

Patients who had at least one SPC consultation (*n* = 210) did not receive tumor-specific treatment in the 30 days prior to death in 83.3% (*n* = 175) of cases, while 16.7% (*n* = 35) did receive tumor-specific treatment. The Pearson´s Chi-square test showed a significant difference between both groups (*p* < 0.001) (Supplementary Table 16). Next, a multivariable binary logistic regression analysis confirmed that patients who received SPC were significantly less likely to have a tumor-specific therapy in the last 30 days prior to death (*p* < 0.001; OR: 0.302; 95% CI: 0.157–0.584) (Table 3). Furthermore, patients with methylation of *MGMT* promoter had the same number of progressions/relapses than patients without (*p* = 0.604) and received the same number of systemic treatments (*p* = 0.245) but were less likely to receive a tumor-specific therapy in the last 30 days before death (*p* = 0.005; OR: 0.375; 95% CI: 0.190–0.739). Age at first diagnosis, sex and last documented KPS showed no significant influence on the receipt of a tumor-specific therapy at the end of life (Table 3).

**Table 3:** Univariable and multivariable binary logistic regression analysis of the influence of clinical characteristics on tumor-specific therapy in the 30 days prior to death (cohort I, *n* = 274)

Variable	Category (*n*)	Univariable binary logistic regression analysis				Multivariable binary logistic regression analysis			
		*p*	OR	Lower 95%-CI	Upper 95%-CI	*p*	OR	Lower 95%-CI	Upper 95%-CI
SPC	No (64)		1.000				1.000		
	Yes (210)	< 0.001	0.333	0.179	0.621	< 0.001	0.302	0.157	0.584
Sex	Male (158)		1.000				1.000		
	Female (116)	0.238	0.698	0.384	1.268	0.873	0.949	0.496	1.813
Age at first diagnosis^2^		0.177	0.983	0.959	1.008	0.216	0.983	0.958	1.010
Last documented KPS^3^		0.066	1.020	0.999	1.041	0.117	1.017	0.996	1.040
*MGMT* promoter methylation	No (157)		1.000				1.000		
	Yes (117)	0.003	0.378	0.198	0.719	0.005	0.375	0.190	0.739

*Abbreviations: OR* Odds Ratio, *CI* Confidence Interval, *SPC* Specialized palliative care, *KPS* Karnofsky Performance Scale

^2^Age, as a continuous variable

^3^KPS, as a categorical variable with 10 point increments

### SPC and survival

Next, we analyzed the association between SPC and survival. The mOS was 16.9 months (95% CI: 14.5–19.3 months) in patients with at least one SPC consultation (*n* = 210) vs. 12.9 months (95% CI: 10.8–15.1 months) in patients without (*n* = 64) (*p* = 0.100) (Supplementary Fig. 1). In exploratory analyses, 148 (70.5%) patients with at least one SPC consultation vs. 37 (57.8%) without were alive at one year and 63 (30.4%) vs. 15 (23.4%) were alive at two years after diagnosis (Supplementary Fig. 1). The multivariable Cox proportional-hazards model showed that receiving at least one SPC consultation is associated with a longer OS (*p* = 0.017, HR: 0.707; 95% CI: 0.532–0.939) (Table 4). Furthermore, the following parameters revealed also a significant correlation with extended OS: female sex (*p* = 0.018, HR: 0.740; 95% CI: 0.577–0.949), complete resection (*p* < 0.001, HR: 0.615; 95% CI: 0.479–0.788) and methylated *MGMT* promoter (*p* < 0.001, HR: 0.379; 95% CI: 0.289–0.496). An older age at diagnosis correlated with a shorter OS (*p* < 0.001, HR: 1.028 per year; 95% CI: 1.016–1.041) (Table 4).

**Table 4:** Cox proportional-hazards regression model of the influence of clinical aspects on OS (cohort I with *n* = 274)

Variable	Category (*n*)	Univariable cox regression analysis				Multivariable cox regression analysis			
		*p*	HR	Lower 95%-CI	Upper 95%-CI	*p*	HR	Lower 95%-CI	Upper 95%-CI
SPC	No (64)		1.000				1.000		
	Yes (210)	0.108	0.794	0.600	1.052	0.017	0.707	0.532	0.939
Sex	Male (158)		1.000				1.000		
	Female (116)	0.079	0.805	0.632	1.025	0.018	0.740	0.577	0.949
Age at first diagnosis^2^		0.003	1.017	1.006	1.028	< 0.001	1.028	1.016	1.041
KPS^3^ at first diagnosis		0.147	0.993	0.094	1.002	0.367	0.995	0.985	1.005
*MGMT* promoter methylation	No (157)		1.000				1.000		
	Yes (117)	< 0.001	0.475	0.369	0.611	< 0.001	0.379	0.289	0.496
Macroscopic complete resection	No (157)		1.000				1.000		
	Yes (117)	0.001	0.669	0.525	0.852	< 0.001	0.615	0.479	0.788

*Abbreviations: OS* Overall survival, *HR* Hazard Ratio, *CI* Confidence Interval, *KPS* Karnofsky Performance Scale, *SPC* Specialized palliative care

^2^Age, as a continuous variable

^3^KPS, as a categorical variable with 10 points increments

The median PFS (mPFS) did not differ significantly between the two groups: mPFS was 7.8 months (95% CI: 6.763–8.941 months) for patients with at least one SPC consultation (*n* = 210) vs. 7.1 months (95% CI: 6.002–8.191 months) for patients without (*n* = 64) (*p* = 0.775) (Supplementary Fig. 2). In exploratory analyses, 53 (25.2%) vs. 17 (26.6%) patients with at least one SPC consultation were relapse/progression-free and/or alive at one year after diagnosis and 17 (8.1%) vs. 5 (7.8%) at two years after diagnosis (Supplementary Fig. 2). In accordance with this, our multivariable Cox proportional-hazards model demonstrated no significant correlation of SPC and PFS (*p* = 0.496). Hence, patients with a complete resection (*p* < 0.001, HR: 0.651; 95% CI: 0.509–0.833) and patients with a methylated *MGMT* promoter (*p* < 0.001, HR: 0.401; 95% CI: 0.306–0.527) had a prolonged PFS (Supplementary Table 17).

## Discussion

Data on the impact of PC on the clinical course and survival of patients with GB are scarce [23]. In this retrospective study, we evaluated different types of PC interventions and their association with treatment patterns and outcomes in a single-center sequential cohort of 274 patients with GB. To our knowledge, this study marks the first complete evaluation of inpatient and outpatient nPPC and SPC interventions in patients with GB.

Whereas the positive impact of PC on symptom control and HR-QoL on patients with a life-limiting disease has been proven in several studies [36], data on the association of PC and survival outcomes, particularly in GB, is scarce and heterogenous. Our retrospective study contributes new scientific insights highlighting an association between SPC and prolonged OS in patients with GB in a real-world setting. In some clinical studies, partly retrospective [29, 30] but also prospective [31], a correlation between PC and reduced survival in patients with GB was observed. There is also data suggesting a positive association of survival outcomes and PC in patients with GB depending on PC referral prior to end of life or during end of life [27]. However, comparability is severely restricted due to methodological differencies: some studies covered different time periods [27, 29–31], PC rates were low [29], or the aim was to investigate early integration of PC [30, 31]. Two published studies in patients with gliomas had severe weaknesses, as they did not exclusively refer to GB and they did not regard both inpatient and outpatient SPC, leading to a underreporting of PC interventions [23, 29]. Other studies focused on comparing early and late integration of PC [27, 29, 31], whereas in our setting, PC supply was included irrespective of timing. Our study does not oppose previous publications on this point, but rather enriches them with a real-world setting.

There are some hypotheses to consider regarding the association between SPC and prolonged survival in this cohort. This could be due to a slower disease progression that allows integration of SPC, while patients with a rapid progression may not have enough time to integrate SPC. However, we hypothesize that improving symptom control and HR-QoL enhances the patients` overall health and may lead to prolonged survival, as shown in other tumor entities [37, 38]. SPC is based on a multidimensional approach with validated assessments and established therapeutic interventions that go beyond the scope of nPPC. Therefore, the inclusion of SPC in standard neuro-oncological care, especially at an early stage of the disease, may help to ensure the most appropriate and effective as well as tolerable administration of cancer therapy.

In our cohort, more than 90% of patients with GB received nPPC, and the indications for nPPC were primarily neurological. This study was conducted in a neuro-oncological setting, so neurological indications were reliably recorded, but indications from other specialties may have been underestimated. Nevertheless, this highlights, that the medical needs of this patient group are not equivalent to those of other tumor entities and require special expertise for their treatment [39]. This also emphasizes that neuro-oncologists must be alerted in view of PC needs and interventions. While PPC is gaining importance in almost all specialist areas and first efforts are being made to define nPPC interventions for neurological patients more precisely [18], nPPC is not yet an integral part in most neurological residency programs [40].

Recent published data reveal a heterogeneous SPC rate in neuro-oncological patients varying between 3 and 40%, depending on the specific tumor entity, the definition of SPC interventions and the method of data collection [26–29]. The level of SPC supply in our patient cohort exceeds the literature references by far, with 76.6% of patients receiving SPC. The reasons for that superior rate must be evaluated in more detail, but they may be, amongst others, that our study was performed in an established certified brain tumor center with a direct affiliation to a university PC center and that we included both inpatient and outpatient settings. In our SPC cohort, 65.7% of patients were integrated into a SPOC team, reflecting the high needs that reach beyond an inpatient stay. The high rate could also be a hint that the implementation of SPC in patients with GB is advancing. Interestingly, amongst the German neuro-oncological certification system that includes a mandatory evaluation of palliative needs from 2023 on, the rates of PC integration vary from 0 to 100% with a median of 26.2% in 2022 [41]. To date, only 35% of neuro-oncological sites in Germany include PC specialists in tumor boards [42]. An increased attendance by PC specialists could reduce the barriers to initiation of SPC and strengthen interdisciplinary cooperation.

Nonetheless, PC integration typically occurs late. The mean time from first diagnosis to start of SPC was 72.4 weeks, with only 8 patients (3.8%) referred to SPC within 8 weeks of diagnosis - contrary to the ASCO guidelines, which recommend early integration within 8 weeks of first diagnosis [43]. A survey among 46 German neuro-oncology sites showed that only 28% of sites refer their patients to PC within first diagnosis [42]. Owing to the rapid progression, high symptom load and reduced life expectancy of patients with GB, the integration of SPC can be unfortunately considered as delayed in most cases.

Several studies proved that SPC is of high benefit for cancer patients [36]. There is recent evidence, that early integration of PC improved QoL, mood and PC problems in patients with GB [31]. However, there are remaining barriers to the initiation of SPC in clinical practice, especially at an early stage of the disease. This may include persistent stigmatization of SPC, leading to the assumption that SPC is not compatible with tumor therapy and is inevitably associated with the end of life [44, 45]. This problem is not only evident in the area of SPC, but also analogously in PC in hospices, which further underscores this point [46, 47]. We hypothesize that an international definition of early integration of SPC and standardized screening assessments for SPC needs is essential. However, cultural and regional specifications may interfere with such a standardization.

Importantly, we demonstrated that patients who received SPC were significantly less likely to have a tumor-specific therapy in the last 30 days prior to death, consistent with the results of previous studies [48]. In accordance to international guidelines, patients with a KPS below 70 are considered ineligible for tumor therapy, at least in a non-curative setting [25]. However, no association between the last documented KPS and the receipt of tumor-specific therapy within the last 30 days of life could be observed. It should be noted that the last documented KPS refers to the most recent tumor relapse/progression in each patient and therefore a temporal variation in this parameter may appear. To this day, simultaneous tumor-specific therapy and PC is not standard practice. A recommendation for early integration of PC clearly argues against this strict separation of tumor-specific therapy and PC, as commonly done in the USA [43], the Netherlands, Australia and New Zealand. We therefore advocate to overcome this strict separation, enabling a simultaneous application of both tumor-specific therapy and PC.

Remarkably, patients with a methylated *MGMT* promoter status are less likely to receive tumor-specific therapy in the last 30 days before death. Although the patient group with *MGMT* promoter methylation in our cohort does not differ in the total number of relapse/progression therapies, the treatment regimens are most likely spread over an extended period, as the disease of these patients is frequently progressing less rapidly [4]. Differences in the extent of treatment and care requirements exist even within the same tumor entity, so further research on treatment factors associated with PC referral is needed.

There are several limitations to this study. This is a single-center analysis in a certified brain tumor center with a direct affiliation to a certified university PC center and a wide rural outreach. Results may vary in multi-center studies or in centers with a more urban population, different sociodemographic, cultural background or without an established brain tumor or PC center. However, the Regensburg Brain Tumor Center represents average quality standards of the German certification system. Furthermore, due to the retrospective design, it is possible that symptoms and PC interventions are not fully recorded. The retrospective assignment of symptoms to categories and subcategories may also be subject to bias, although we have performed repeated validity checks. However, the high rate of both nPPC and SPC suggests that the documentation is largely complete. Furthermore, recording PPC by primary care physicians was not possible owing to a lack of documentation. However, we consciously focused on collecting data on nPPC, as this is a special feature of care for patients with GB in a brain tumor center setting. As a result of the retrospective design, the timing of data collection, such as the last documented KPS, varies according to the individual clinical course, which may lead to a potential inaccuracy. Patients who were still alive at the time of the last data assessment were excluded from the analysis for methodological reasons, which could, however, lead to bias. Lastly, the retrospective nature of the study did not allow us to include caregivers. Future research should address the problems of caregivers and their potential for the patient’s disease trajectory.

This study also contains important strengths. We analyzed a large and homogeneous cohort of patients with GB, reclassified in the WHO classification from 2021, who were treated at a major brain tumor site over a period of 10 years. By collecting data not only from medical records, but also through personal queries of SPOC teams and primary care physicians, it was possible to achieve a high level of data quality. In addition, we collected data from the inpatient and outpatient setting, and data from nPPC and SPC, making this cohort unique in literature.

As a major result, we show a positive association between OS and SPC in our Kaplan Meier estimation as well as multivariable regression analysis. The next steps will be to verify our data in an independent cohort and to develop a prospective strategy for the early and full implementation of PC for patients with GB.

## Conclusion

We conclude that patients with GB benefit from the implementation of both nPPC and SPC. As this is not standard for all patients with GB, utmost efforts should be made to incorporate PC into the early care of every patient with GB within a standardized framework. This study further highlights the importance of future research on a cross-sectional approach to identify PC needs as well as the kind and frequency of interventions in this highly burdened patient group, including caregivers. Demographic changes and more effective tumor therapies may lead to an increase in long-term disease trajectories and less rapid progression, which will make PC even more relevant in the future. Therefore, further research should focus on the ideal timing of PC integration and on identifying patient groups who are most likely to benefit from PC.

## Supplementary Information

Supplementary Material 1

## Data Availability

The datasets used and/or analysed during the current study are available from the corresponding author on reasonable request.

## Abbreviations

GB: Glioblastoma
IDHwt: Isocitrate dehydrogenase wild type
OS: Overall survival
*MGMT*: O-6-methylguanine-DNA methyltransferase
KPS: Karnofsky performance status scale
HR-QoL: Health-related quality of life
PC: Palliative care
PPC: Primary palliative care
SPC: Specialized palliative care
nPPC: Neuro-oncologically focused primary palliative care
SPOC: Specialized palliative outpatient care
PCU: Palliative care unit
PFS: Progression-free survival
mOS: Median overall survival
mPFS: Median progression-free survival

## References

[1] Louis DN, Perry A, Wesseling P, Brat DJ, Cree IA, Figarella-Branger D et al. The 2021 WHO Classification of Tumors of the Central Nervous System: a summary. Neuro-oncology 2021; 23(8):1231–51. Available from: URL: https://pubmed.ncbi.nlm.nih.gov/34185076/. 10.1093/neuonc/noab106 PMC8328013 34185076

[2] Price M, Ballard CAP, Benedetti JR, Kruchko C, Barnholtz-Sloan JS, Ostrom QT. CBTRUS Statistical Report: Primary Brain and Other Central Nervous System Tumors Diagnosed in the United States in 2018–2022. Neuro-oncology. 2025; 27(Supplement_4):iv1-iv66. Available from: URL: https://pubmed.ncbi.nlm.nih.gov/41092086/10.1093/neuonc/noaf194 PMC12527013 41092086

[3] Hegi ME, Diserens A-C, Gorlia T, Hamou M-F, de Tribolet N, Weller M, et al. MGMT gene silencing and benefit from temozolomide in glioblastoma. N Engl J Med. 2005;352(10):997–1003. Available from: URL:. https://pubmed.ncbi.nlm.nih.gov/15758010/10.1056/NEJMoa043331 15758010

[4] Herrlinger U, Tzaridis T, Mack F, Steinbach JP, Schlegel U, Sabel M, et al. Lomustine-temozolomide combination therapy versus standard Temozolomide therapy in patients with newly diagnosed glioblastoma with methylated MGMT promoter (CeTeG/NOA-09): a randomised, open-label, phase 3 trial. Lancet. 2019;393(10172):678–88. 30782343 10.1016/S0140-6736(18)31791-4

[5] Stupp R, Taillibert S, Kanner A, Read W, Steinberg D, Lhermitte B et al. Effect of Tumor-Treating Fields Plus Maintenance Temozolomide vs Maintenance Temozolomide Alone on Survival in Patients With Glioblastoma: A Randomized Clinical Trial. JAMA. 2017; 318(23):2306–16. Available from: URL. https://pubmed.ncbi.nlm.nih.gov/29260225/10.1001/jama.2017.18718 PMC5820703 29260225

[6] Armstrong TS, Vera-Bolanos E, Acquaye AA, Gilbert MR, Ladha H, Mendoza T. The symptom burden of primary brain tumors: evidence for a core set of tumor- and treatment-related symptoms. Neuro-oncology. 2016; 18(2):252–60. Available from: URL. https://pubmed.ncbi.nlm.nih.gov/26289592/10.1093/neuonc/nov166 PMC4724180 26289592

[7] Peeters MCM, Dirven L, Koekkoek JAF, Gortmaker EG, Fritz L, Vos MJ et al. Prediagnostic symptoms and signs of adult glioma: the patients’ view. Journal of neuro-oncology. 2020; 146(2):293–301. Available from: URL. https://pubmed.ncbi.nlm.nih.gov/31894516/10.1007/s11060-019-03373-y 31894516

[8] Coomans MB, Dirven L, Aaronson NK, Baumert BG, van den Bent M, Bottomley A et al. Symptom clusters in newly diagnosed glioma patients: which symptom clusters are independently associated with functioning and global health status? Neuro-oncology. 2019; 21(11):1447–57. Available from: URL. https://pubmed.ncbi.nlm.nih.gov/31682733/10.1093/neuonc/noz118 PMC6827824 31682733

[9] Lin E, Rosenthal MA, Eastman P, Le BH. Inpatient palliative care consultation for patients with glioblastoma in a tertiary hospital. Internal Medicine Journal. 2013; 43(8):942–5. Available from: URL. https://pubmed.ncbi.nlm.nih.gov/23919337/10.1111/imj.12211 23919337

[10] Sizoo EM, Braam L, Postma TJ, Pasman HRW, Heimans JJ, Klein M et al. Symptoms and problems in the end-of-life phase of high-grade glioma patients. Neuro-oncology. 2010; 12(11):1162–6. Available from: URL: https://academic.oup.com/neuro-oncology/article/12/11/1162/1136696 10.1093/neuonc/nop045 PMC3098016 20511193

[11] Walbert T, Schultz L, Mikkelsen T, Snyder JM, Phillips J, Fortunato JT. Prospective assessment of end-of-life symptoms and quality of life in patients with high-grade glioma. Neurooncol Pract. 2024; 11(6):733–9. Available from: URL: https://pubmed.ncbi.nlm.nih.gov/39554791/10.1093/nop/npae056 PMC11567736 39554791

[12] Rooney AG, McNamara S, Mackinnon M, Fraser M, Rampling R, Carson A et al. The frequency, longitudinal course, clinical associations, and causes of emotional distress during primary treatment of cerebral glioma. Neuro-oncology. 2013; 15(5):635–43. Available from: URL: https://pubmed.ncbi.nlm.nih.gov/23444258/10.1093/neuonc/not009 PMC3635525 23444258

[13] Bergo E, Lombardi G, Guglieri I, Capovilla E, Pambuku A, Zagone V. Neurocognitive functions and health-related quality of life in glioblastoma patients: a concise review of the literature. European journal of cancer care. 2019; 28(1):e12410. Available from: URL: https://onlinelibrary.wiley.com/doi/10.1111/ecc.12410 10.1111/ecc.12410 26531122

[14] Halkett GKB, Lobb EA, Rogers MM, Shaw T, Long AP, Wheeler HR et al. Predictors of distress and poorer quality of life in High Grade Glioma patients. Patient education and counseling. 2015; 98(4):525–32. Available from: URL: https://pubmed.ncbi.nlm.nih.gov/25638306/10.1016/j.pec.2015.01.002 25638306

[15] van Oorschot B, Jentschke E, Kessler AF, Wittig A, Kamp M. Dringende Notwendigkeit für strukturiertes Symptom- und Belastungsscreening bei Patienten mit primären und sekundären Hirntumoren. Onkologie. 2023; 29(11):984–90. Available from: URL: https://link.springer.com/article/10.1007/s00761-023-01417-7

[16] Quill TE, Abernethy AP. Generalist plus specialist palliative care–creating a more sustainable model. N Engl J Med. 2013;368(13):1173–5. 23465068 10.1056/NEJMp1215620

[17] Rhee JY, Strander S, Podgurski A, Chiu D, Brizzi K, Forst DA. Palliative Care in Neuro-oncology: an Update. Current neurology and neuroscience reports. 2023; 23(11):645–56. Available from: URL: https://pubmed.ncbi.nlm.nih.gov/37751050/10.1007/s11910-023-01301-2 37751050

[18] Harrison DJ, Wu E, Singh R, Ghaith S, Suarez-Meade P, Brown NJ et al. Primary and Specialist Palliative Care in Neurosurgery: A Narrative Review and Bibliometric Analysis of Glioblastoma and Stroke. World neurosurgery. 2023; 180:e250-e257. Available from: URL: https://pubmed.ncbi.nlm.nih.gov/37739173/10.1016/j.wneu.2023.09.048 37739173

[19] DGP. Positionspapier der AG Stationäre Versorgung zur aktuellen Entwicklung von Qualität 2011.

[20] Gesell D, Hodiamont F, Bausewein C, Koller D. Accessibility to specialist palliative care services in Germany: a geographical network analysis. BMC health services research. 2023; 23(1):786. Available from: URL: https://pubmed.ncbi.nlm.nih.gov/37488579/10.1186/s12913-023-09751-7 PMC10364400 37488579

[21] Deutsche Gesellschaft für Palliativmedizin e.V. (DGP). Erweiterte S3-Leitlinie Palliativmedizin für Patienten mit einer nicht heilbaren Krebserkrankung: AWMF Leitlinienregister; 2021 [cited 2024 Jul 12]. Available from: URL: https://register.awmf.org/de/leitlinien/detail/128-001OL

[22] Zertifizierungskommission Onkologische Zentren. Erhebungsbogen für Onkologische Spitzenzentren und Onkologische Zentren: Nationales Zertifizierungsprogramm Krebs. 2023. Available from: URL: https://www.krebsgesellschaft.de/zertdokumente.html

[23] Byrne A, Torrens-Burton A, Sivell S, Moraes FY, Bulbeck H, Bernstein M et al. Early palliative interventions for improving outcomes in people with a primary malignant brain tumour and their carers. The Cochrane database of systematic reviews. 2022; 1(1):CD013440. Available from: URL: https://pubmed.ncbi.nlm.nih.gov/34988973/10.1002/14651858.CD013440.pub2 PMC8733789 34988973

[24] Feng C, Wu Y, Gao L, Guo X, Wang Z, Xing B. Publication Landscape Analysis on Gliomas: How Much Has Been Done in the Past 25 Years? Frontiers in Oncology. 2019; 9:1463. Available from: URL: https://www.ncbi.nlm.nih.gov/pmc/articles/PMC6988829/10.3389/fonc.2019.01463 PMC6988829 32038995

[25] Pace A, Dirven L, Koekkoek JAF, Golla H, Fleming J, Rudà R et al. European Association for Neuro-Oncology (EANO) guidelines for palliative care in adults with glioma. The Lancet. Oncology. 2017; 18(6):e330-e340. Available from: URL: https://www.sciencedirect.com/science/article/pii/S1470204517303455 10.1016/S1470-2045(17)30345-5 28593859

[26] Fink L, van Oorschot B, von Saß C, Dibué M, Foster M-T, Golla H et al. Palliative care for in-patient malignant glioma patients in Germany. Journal of neuro-oncology. 2024; 167(2):323–38. Available from: URL. https://pubmed.ncbi.nlm.nih.gov/38506960/10.1007/s11060-024-04611-8 PMC11023986 38506960

[27] Crooms RC, Taylor JW, Jette N, Morgenstern R, Agarwal P, Goldstein NE, et al. Palliative care referral across the disease trajectory in high-grade glioma. J Neurooncol. 2023;163(1):249–59. 37209290 10.1007/s11060-023-04338-y PMC10546385

[28] Wu A, Ruiz Colón G, Aslakson R, Pollom E, Patel CB. Palliative Care Service Utilization and Advance Care Planning for Adult Glioblastoma Patients: A Systematic Review. Cancers. 2021; 13(12). Available from: URL: https://pubmed.ncbi.nlm.nih.gov/34201260/10.3390/cancers13122867 PMC8228109 34201260

[29] Pando A, Patel AM, Choudhry HS, Eloy JA, Goldstein IM, Liu JK. Palliative Care Effects on Survival in Glioblastoma: Who Receives Palliative Care? World neurosurgery. 2023; 170:e847-e857. Available from: URL: https://www.sciencedirect.com/science/article/pii/S1878875022016953 10.1016/j.wneu.2022.11.143 36481442

[30] Wu A, Ugiliweneza B, Wang D, Hsin G, Boakye M, Skirboll S. Trends and outcomes of early and late palliative care consultation for adult patients with glioblastoma: A SEER-Medicare retrospective study. Neurooncol Pract. 2022; 9(4):299–309. Available from: URL: https://academic.oup.com/nop/article/9/4/299/6555764 10.1093/nop/npac026 PMC9290893 35859543

[31] Golla H, Nettekoven C, Hellmich M, Appelmann I, Bausewein C, Becker G et al. Early Palliative Care for Patients with Glioblastoma: A randomized phase lll clinical trial (EPCOG). Neuro-oncology 2025. Available from: URL: https://academic.oup.com/neuro-oncology/advance-article/doi/10.1093/neuonc/noaf230/8280414?login=false 10.1093/neuonc/noaf230 PMC12962646 41071052

[32] Koekkoek JAF, van der Meer PB, Pace A, Hertler C, Harrison R, Leeper HE et al. Palliative care and end-of-life care in adults with malignant brain tumors. Neuro-oncology. 2023; 25(3):447–56. Available from: URL: https://pmc.ncbi.nlm.nih.gov/articles/PMC10013651/10.1093/neuonc/noac216 PMC10013651 36271873

[33] Stupp R, Mason WP, van den Bent MJ, Weller M, Fisher B, Taphoorn MJB et al. Radiotherapy plus concomitant and adjuvant temozolomide for glioblastoma. The New England journal of medicine. 2005; 352(10):987–96. Available from: URL: https://pubmed.ncbi.nlm.nih.gov/15758009/10.1056/NEJMoa043330 15758009

[34] Weller M, van den Bent M, Preusser M, Le Rhun E, Tonn JC, Minniti G et al. EANO guidelines on the diagnosis and treatment of diffuse gliomas of adulthood. Nature reviews. Clinical oncology. 2021; 18(3):170–86. Available from: URL: https://pubmed.ncbi.nlm.nih.gov/33293629/10.1038/s41571-020-00447-z PMC7904519 33293629

[35] Medizinischer Dienst des Spitzenverbandes Bund der Krankenkassen e.V. (MDS). Begutachtungsanleitung Spezialisierte ambulante Palliativversorgung (SAPV) und stationäre Hospizversorgung: Richtlinie des GKV-Spitzenverbandes nach § 282 SGB V. 2019. Available from: URL: https://www.gkv-spitzenverband.de/media/dokumente/krankenversicherung_1/hospiz_palliativversorgung/20190213_BGA_SAPV_und_stationare_Hospizversorgung_final.pdf

[36] Kavalieratos D, Corbelli J, Di Zhang, Dionne-Odom JN, Ernecoff NC, Hanmer J et al. Association Between Palliative Care and Patient and Caregiver Outcomes: A Systematic Review and Meta-analysis. JAMA. 2016; 316(20):2104–14. Available from: URL: https://pubmed.ncbi.nlm.nih.gov/27893131/10.1001/jama.2016.16840 PMC5226373 27893131

[37] Temel JS, Greer JA, Muzikansky A, Gallagher ER, Admane S, Jackson VA et al. Early palliative care for patients with metastatic non-small-cell lung cancer. The New England journal of medicine. 2010; 363(8):733–42. Available from: URL: https://pubmed.ncbi.nlm.nih.gov/20818875/10.1056/NEJMoa1000678 20818875

[38] Bakitas MA, Tosteson TD, Li Z, Lyons KD, Hull JG, Li Z et al. Early Versus Delayed Initiation of Concurrent Palliative Oncology Care: Patient Outcomes in the ENABLE III Randomized Controlled Trial. Journal of clinical oncology: official journal of the American Society of Clinical Oncology. 2015; 33(13):1438–45. Available from: URL: https://pubmed.ncbi.nlm.nih.gov/25800768/10.1200/JCO.2014.58.6362 PMC4404422 25800768

[39] Crooms RC, Nnemnbeng JF, Taylor JW, Goldstein NE, Gorbenko K, Vickrey BG. Clinician perspectives on integrating neuro-oncology and palliative care for patients with high-grade glioma. Neuro Oncol Pract. 2024. 10.1093/nop/npae022. 10.1093/nop/npae022 PMC11241354 39006519

[40] Creutzfeldt CJ, Robinson MT, Holloway RG. Neurologists as primary palliative care providers: Communication and practice approaches. Neurology. Clinical practice. 2016; 6(1):40–8. Available from: URL: https://pubmed.ncbi.nlm.nih.gov/26918202/10.1212/CPJ.0000000000000213 PMC4753829 26918202

[41] Onkozert. Jahresberichte O. 2025 [cited 2025 Jul 16]. Available from: URL: https://www.onkozert.de/info/jahresberichte/

[42] Lawson McLean AC, Lawson McLean A, Ernst T, Forster M-T, Freyschlag C, Gempt J, et al. Benchmarking palliative care practices in neurooncology: a german perspective. J Neurooncol. 2024. 10.1007/s11060-024-04674-7. 38696050 10.1007/s11060-024-04674-7 PMC11147867

[43] Ferrell BR, Temel JS, Temin S, Alesi ER, Balboni TA, Basch EM, Integration of Palliative Care Into Standard Oncology Care. American Society of Clinical Oncology Clinical Practice Guideline Update. Journal of clinical oncology: official journal of the American Society of Clinical Oncology. : 2017; 35(1):96–112. Available from: URL: https://pubmed.ncbi.nlm.nih.gov/28034065/10.1200/JCO.2016.70.1474 28034065

[44] Parajuli J, Hupcey JE. A systematic review on barriers to palliative care in oncology. Am J Hosp Palliat Care. 2021;38(11):1361–77. 33412898 10.1177/1049909120983283

[45] Vierhout M, Daniels M, Mazzotta P, Vlahos J, Mason WP, Bernstein M. The views of patients with brain cancer about palliative care: a qualitative study. Curr Oncol. 2017; 24(6):374–82. Available from: URL: https://www.mdpi.com/1718-7729/24/6/3712 10.3747/co.24.3712 PMC5736479 29270049

[46] Kaiser U, Vehling-Kaiser U, Kück F, Mechie N-C, Hoffmann A, Kaiser F. Use of symptom-focused oncological cancer therapies in hospices: a retrospective analysis. BMC palliative care. 2020; 19(1):140. Available from: URL: https://bmcpalliatcare.biomedcentral.com/articles/10.1186/s12904-020-00648-4 10.1186/s12904-020-00648-4 PMC7488695 32919468

[47] Kaiser U, Vehling-Kaiser U, Hoffmann A, Kaiser F. Inpatient Hospices in Germany: Medical Care Situation and Use of Supportive Oncological Therapies for Symptom Control in Tumor Patients. Palliative Medicine Reports. 2022; 3(1):169–80. Available from: URL: https://pmc.ncbi.nlm.nih.gov/articles/PMC9438444/10.1089/pmr.2022.0026 PMC9438444 36059908

[48] Kubendran S, Schockett E, Jackson E, Huynh-Le MP, Roberti F, Rao YJ et al. Trends in inpatient palliative care use for primary brain malignancies. Support Care Cancer. 2021; 29(11):6625–32. Available from: URL: https://link.springer.com/article/10.1007/s00520-021-06255-0 10.1007/s00520-021-06255-0 33945016

