# Supplementary material for: Palliative care interventions and outcome in patients with glioblastoma – a retrospective, single-center study
Source: BMC Palliat Care. 2026 Jan 16;25:29. doi: 10.1186/s12904-026-01987-4 (PMC12836934; doi:10.1186/s12904-026-01987-4)
Supplement: Supplementary file 1 — Supplementary Material 1 [file 12904_2026_1987_MOESM1_ESM.docx]

**Palliative Care Interventions and Outcome in Patients with Glioblastoma – a retrospective, single-center Study**

Lisa-Marie Lind^1^, Anna Fischl^1^, Elisabeth Goettl^2,3^, Wolfgang Herr^4^, Ulrich Kaiser^5^, Oliver Koelbl^6^, Ralf Linker^1^, Julia Maurer^2,3^, Markus J. Riemenschneider^7^, Nils-Ole Schmidt^3,8^, Martin Proescholdt^8^, Tobias Pukrop^4^, Peter Hau^1,3^, Michael Rechenmacher^4,9^, Elisabeth Bumes^1^

| Symptom cluster | Subcategory | Medical intervention |
| --- | --- | --- |
| Pain |  | - start or increase of opiate medication - combination of at least two pain medications - start or increase of neuropathic pain medication |
| Neurological symptoms | Seizure | - more than one medication for seizure prophylaxis - change of seizure-suppressing medication |
|  | Aphasia or neurocognition | - initiation of speech therapy - neurocognitive training - treatment of dysphagia |
|  | Other neurologic symptoms | e.g. corticosteroids for cerebral edema |
| Psychiatric/psychological symptoms | Depression or lack of motivation | start or increase of antidepressant medication |
|  | Delirium or hallucinations | start or increase of neuroleptic medication |
|  | Change of personality | - symptom control of aggressiveness - symptom control of cognitive disorders - psychosocial support |
|  | Other psychiatric symptoms | e.g. anxiolytic medication, sleep medication |
| Cardiac/respiratory symptoms |  | e.g. opiate for respiratory distress |
| Gastrointestinal symptoms | Nausea or emesis | start or change of antiemetic therapy |
|  | Diarrhoea/constipation | - start or change of peristaltic inhibitors - start or change of laxative measures |
| Wounds |  | e.g. treatment of decubitus |
| Urogenital symptoms |  | e.g. anticholinergic agent in neurogenic disorders of micturition |
| Other symptoms |  | e.g. therapy for sepsis, high-calorie drinks for cachexia, baclofen for cramps, psychosocial support of caregivers |

**Supplementary Table 1** Symptoms at the start of nPPC and indications for nPPC.

**Abbreviations:** nPPC, neurooncologically-focused primary palliative care.

| Indication | Definition |
| --- | --- |
| Symptoms of complex palliative care | - Severe pain symptoms - Severe neurological/psychiatric/ psychological symptoms - Severe cardiac/respiratory symptoms - Severe gastrointestinal symptoms - Severe ulcerating/exulcerating wounds/tumors - Severe urogenital symptoms - Other severe symptoms |
| Consultation at first diagnosis | Consultation by PC specialist within 8 weeks of first diagnosis |
| Therapy goal setting | Consultation by a PC specialist for therapy goal setting |
| Care coordination | Evaluation of individual care coordination |
| Other indication | e.g. Psychosocial support of caregiver  e.g. Domestic support |

**Supplementary Table 2** Indications for inpatient palliative consultation.

**Abbreviations:** PC, palliative care.

| Symptoms of complex palliative care | - Severe pain symptoms - Severe neurological/psychiatric/   psychological symptoms   - Severe cardiac/respiratory symptoms - Severe gastrointestinal symptoms - Severe ulcerating/exulcerating wounds/tumors - Severe urogenital symptoms - Other severe symptoms |
| --- | --- |

**Supplementary Table 3** Indications for SPOC and PCU.

**Abbreviations:** SPOC, specialized palliative outpatient care; PCU, palliative care unit.

**Supplementary Table 4** STROBE Statement—Checklist of items that should be included in reports of cohort studies

|  | Item No | Recommendation |
| --- | --- | --- |
| **Title and abstract** | 1 | *(*a) Indicate the study’s design with a commonly used term in the title or the abstract  Single-center, retrospective cohort study as stated in the Abstract on page 2 and Materials and Methods on page 5. |
|  |  | (*b*) Provide in the abstract an informative and balanced summary of what was done and what was found  Provided in Abstract on page 2. |
| Introduction | | |
| Background/rationale | 2 | Explain the scientific background and rationale for the investigation being reported  Included in the Introduction on pages 3 and 4. |
| Objectives | 3 | State specific objectives, including any prespecified hypotheses  Objectives are the receipt of tumor-specific therapy within the last 30 days prior to death, the overall and progression-free survival as included in the Introduction on page 7. |
| Methods | | |
| Study design | 4 | Present key elements of study design early in the paper  Provided in the Materials and Methods on pages 4 and 5. |
| Setting | 5 | Describe the setting, locations, and relevant dates, including periods of recruitment, exposure, follow-up, and data collection  Included in the Materials and Methods on pages 4 and 5. |
| Participants | 6 | (*a*) Give the eligibility criteria, and the sources and methods of selection of participants. Describe methods of follow-up  Included in the Materials and Methods on pages 4, 5 and 6. |
|  |  | (*b*) For matched studies, give matching criteria and number of exposed and unexposed  Not applicable. |
| Variables | 7 | Clearly define all outcomes, exposures, predictors, potential confounders, and effect modifiers. Give diagnostic criteria, if applicable  Included in the Materials and Methods on pages 5 and 6. |
| Data sources/ measurement | 8* | For each variable of interest, give sources of data and details of methods of assessment (measurement). Describe comparability of assessment methods if there is more than one group  Included in the Materials and Methods on pages 5 and 6. |
| Bias | 9 | Describe any efforts to address potential sources of bias  *“[…] we have performed repeated validity checks”* (p. 18) |
| Study size | 10 | Explain how the study size was arrived at  Patients with GB, IDHwt, who were registered in the local tumor registry between January 2014 and February 2024 and filed in our hospital data management system. Included in the Materials and Methods on pages 4 and 5. |
| Quantitative variables | 11 | Explain how quantitative variables were handled in the analyses. If applicable, describe which groupings were chosen and why  Included in the Materials and Methods on page 7. |
| Statistical methods | 12 | (*a*) Describe all statistical methods, including those used to control for confounding  Detailed description in the Materials and Methods on page 7. |
|  |  | (*b*) Describe any methods used to examine subgroups and interactions  Not applicable. |
|  |  | (*c*) Explain how missing data were addressed  Identified as “no data available”. |
|  |  | (*d*) If applicable, explain how loss to follow-up was addressed  Not applicable. |
|  |  | (*e*) Describe any sensitivity analyses  Not applicable. |
| Results | | |
| Participants | 13* | (a) Report numbers of individuals at each stage of study—eg numbers potentially eligible, examined for eligibility, confirmed eligible, included in the study, completing follow-up, and analysed  The inclusion criteria are described in the Materials and Methods on pages 5 and 6. |
|  |  | (b) Give reasons for non-participation at each stage  The exclusion criteria are described in the Materials and Methods on pages 5 and 6. |
|  |  | (c) Consider use of a flow diagram  Provided in the introduction, in Figure 1 on page 6. |
| Descriptive data | 14* | (a) Give characteristics of study participants (eg demographic, clinical, social) and information on exposures and potential confounders  Included in the Results on pages 8 and 9. |
|  |  | (b) Indicate number of participants with missing data for each variable of interest  As shown in the respective tables and supplementary tables. |
|  |  | (c) Summarise follow-up time (eg, average and total amount)  Not applicable. |
| Outcome data | 15* | Report numbers of outcome events or summary measures over time  Included in the Results on page 8 to 13. |
| Main results | 16 | (*a*) Give unadjusted estimates and, if applicable, confounder-adjusted estimates and their precision (eg, 95% confidence interval). Make clear which confounders were adjusted for and why they were included  Confounder-adjusted estimates are included in the Results on page 11 to 13. |
|  |  | (*b*) Report category boundaries when continuous variables were categorized  Not applicable. |
|  |  | (*c*) If relevant, consider translating estimates of relative risk into absolute risk for a meaningful time period  Not applicable. |
| Other analyses | 17 | Report other analyses done—eg analyses of subgroups and interactions, and sensitivity analyses  Not applicable. |
| Discussion | | |
| Key results | 18 | Summarise key results with reference to study objectives  Addressed in the Discussion on page 13 to 16. |
| Limitations | 19 | Discuss limitations of the study, taking into account sources of potential bias or imprecision. Discuss both direction and magnitude of any potential bias  Addressed in the Discussion on page 13 to 17. |
| Interpretation | 20 | Give a cautious overall interpretation of results considering objectives, limitations, multiplicity of analyses, results from similar studies, and other relevant evidence  Addressed in the Discussion on page 13 to 17. |
| Generalisability | 21 | Discuss the generalisability (external validity) of the study results  Addressed in the Discussion on page 13 to 17. |
| Other information | | |
| Funding | 22 | Give the source of funding and the role of the funders for the present study and, if applicable, for the original study on which the present article is based  No funding. |

*Give information separately for exposed and unexposed groups.

**Note:** An Explanation and Elaboration article discusses each checklist item and gives methodological background and published examples of transparent reporting. The STROBE checklist is best used in conjunction with this article (freely available on the Web sites of PLoS Medicine at http://www.plosmedicine.org/, Annals of Internal Medicine at http://www.annals.org/, and Epidemiology at http://www.epidem.com/). Information on the STROBE Initiative is available at <http://www.strobe-statement.org>.

**Supplementary Table 5** Demographic and clinical aspects of cohort III (*n* = 251).

|  | | Valid Number | Percent |
| --- | --- | --- | --- |
| Age at first diagnosis  (years) | 20.0 – 49.9 | 31 | 12.4% |
|  | 50.0 – 59.9 | 85 | 33.9% |
|  | 60.0 – 69.9 | 86 | 34.2% |
|  | ≥ 70 | 49 | 19.5% |
| Sex | Male | 146 | 58.2% |
|  | Female | 105 | 41.8% |
| KPS at start of nPPC | < 70 | 26 | 10.4% |
|  | 70 – 80 | 94 | 37.5% |
|  | 90 – 100 | 120 | 47.7% |
|  | No data available | 11 | 4.4% |
| Number of relapses/progressions  at start of nPPC | 0 | 177 | 70.5% |
|  | > 1 | 74 | 29.5% |
| Start of nPPC | Within 3 weeks before diagnosis | 21 | 8.4% |
|  | Within the first week of diagnosis | 51 | 20.3% |
|  | Within 1 – 10 weeks after diagnosis | 59 | 23.5% |
|  | Within 11 – 30 weeks after diagnosis | 51 | 20.3% |
|  | Within > 30 weeks after diagnosis | 69 | 27.5% |
| Start of nPPC before death in months | < 7 | 85 | 33.9% |
|  | 7 – 12 | 57 | 22.7% |
|  | > 12 | 109 | 43.4% |
| Place of death | At home | 102 | 40.6% |
|  | PCU | 45 | 17.9% |
|  | Hospice | 28 | 11.2% |
|  | Hospital | 30 | 12.0% |
|  | Nursing home | 11 | 4.4% |
|  | No data available | 35 | 13.9% |
|  | Total | 251 | 100% |

**Abbreviations:** KPS, Karnofsky Performance Scale; nPPC, neurooncologically-focused primary

palliative care; PCU, palliative care unit.

**Supplementary Table 6** Demographic and clinical aspects of cohort IV (*n* = 210).

|  | | Valid Number | Percent |
| --- | --- | --- | --- |
| Age at first diagnosis  (years) | 20.0 – 49.9 | 25 | 11.9% |
|  | 50.0 – 59.9 | 71 | 33.8% |
|  | 60.0 – 69.9 | 76 | 36.2% |
|  | ≥ 70 | 38 | 18.1% |
| Sex | Male | 118 | 56.2% |
|  | Female | 92 | 43.8% |
| KPS at start of SPC | < 70 | 66 | 31.4% |
|  | 70 – 80 | 64 | 30.5% |
|  | 90 – 100 | 10 | 4.8% |
|  | No data available | 70 | 33.3% |
| Number of relapses/progressions  at start of SPC | 0 | 40 | 19.0% |
|  | 1 | 44 | 21.0% |
|  | 2 | 64 | 30.5% |
|  | > 2 | 62 | 29.5% |
| Start of SPC | Within 8 weeks of diagnosis | 8 | 3.8% |
|  | Within 9 – 50 weeks after diagnosis | 77 | 36.7% |
|  | Within 51 – 100 weeks after diagnosis | 76 | 36.2% |
|  | Within > 100 weeks after diagnosis | 49 | 23.3% |
| Start of SPC before death in weeks | < 3 | 64 | 30.5% |
|  | 3 – 10 | 78 | 37.1% |
|  | > 10 | 68 | 32.4% |
| Place of death | At home | 98 | 46.7% |
|  | PCU | 50 | 23.8% |
|  | Hospice | 31 | 14.8% |
|  | Hospital | 11 | 5.2% |
|  | Nursing home | 8 | 3.8% |
|  | No data available | 12 | 5.7% |
| Overall inpatient SPC | No | 81 | 38.6% |
|  | Yes | 129 | 61.4% |
| Inpatient palliative consultation | No | 130 | 61.9% |
|  | Yes | 80 | 38.1% |
| PCU | No | 128 | 61.0% |
|  | Yes | 82 | 39.0% |
| SPOC | No | 72 | 34.3% |
|  | Yes | 138 | 65.7% |
| Hospice | No | 179 | 85.2% |
|  | Yes | 31 | 14.8% |
|  | Total | 210 | 100% |

**Abbreviations:** KPS, Karnofsky Performance Scale; SPC, specialized palliative care; PCU, palliative care unit; SPOC, specialized palliative outpatient care.

**Supplementary Table 7** Symptoms at the start of nPPC (cohort III, *n* = 251).

|  | Valid Number | Percent |
| --- | --- | --- |
| Pain | 14 | 5.6% |
| Seizure | 55 | 21.9% |
| Aphasia or neurocognition | 14 | 5.6% |
| Other neurologic symptoms | 122 | 48.6% |
| Depression or lack of motivation | 29 | 11.6% |
| Delirium or hallucinations | 4 | 1.6% |
| Change of personality | 4 | 1.6% |
| Other psychiatric symptoms | 6 | 2.4% |
| Nausea or emesis | 3 | 1.2% |
| Total | 251 | 100% |

**Abbreviations:** nPPC, neurooncologically-focused primary palliative care.

**Supplementary Table 8** Indications of nPPC (cohort III, *n* = 251).

|  |  |  | Valid Number | Percent |
| --- | --- | --- | --- | --- |
| Pain |  | No | 188 | 74.9% |
|  |  | Yes | 63 | 25.1% |
| Overall neurological symptoms |  | No | 10 | 4.0% |
|  |  | Yes | 241 | 96.0% |
| Seizure |  | No | 128 | 51.0% |
|  |  | Yes | 123 | 49.0% |
| Aphasia or neurocognition |  | No | 206 | 82.1% |
|  |  | Yes | 45 | 17.9% |
| Other neurologic symptoms |  | No | 27 | 10.8% |
|  |  | Yes | 224 | 89.2% |
| Overall psychiatric symptoms |  | No | 113 | 45.0% |
|  |  | Yes | 138 | 55.0% |
| Depression or lack of motivation |  | No | 144 | 57.4% |
|  |  | Yes | 107 | 42.6% |
| Delirium or hallucinations |  | No | 219 | 87.3% |
|  |  | Yes | 32 | 12.7% |
| Change of personality |  | No | 241 | 96.0% |
|  |  | Yes | 10 | 4.0% |
| Other psychiatric symptoms |  | No | 211 | 84.1% |
|  |  | Yes | 40 | 15.9% |
| Neurological and/or psychiatric symptoms |  | No | 10 | 4.0% |
|  |  | Yes | 241 | 96.0% |
| Cardiac and respiratory symptoms |  | No | 250 | 99.6% |
|  |  | Yes | 1 | 0.4% |
| Gastrointestinal symptoms |  | No | 212 | 84.5% |
|  |  | Yes | 39 | 15.5% |
| Wounds |  | No | 251 | 100.0% |
|  |  | Yes | 0 | 0.0% |
| Urogenital symptoms |  | No | 250 | 99.6% |
|  |  | Yes | 1 | 0.4% |
| Other symptoms |  | No | 249 | 99.2% |
|  |  | Yes | 2 | 0.8% |
|  |  | Total | 251 | 100.0% |

**Abbreviations:** nPPC, neurooncologically-focused primary palliative care.

**Supplementary Table 9** Characteristics of patients with inpatient palliative consultation (cohort IV, *n* = 210).

|  | | Inpatient palliative consultation | | | | | |  |
| --- | --- | --- | --- | --- | --- | --- | --- | --- |
|  |  | No | | Yes | | Total | | X^2^ |
|  |  | n | (%) | n | (%) | n | (%) | *p* |
| Sex | Male | 72 | 55.4% | 46 | 57.5% | 118 | 56.2% | .764 |
|  | Female | 58 | 44.6% | 34 | 42.5% | 92 | 43.8% |  |
|  | Total | 130 | 100.0% | 80 | 100.0% | 210 | 100.0% |  |
| Age at diagnosis in years | 20.0 – 49.9 | 16 | 12.3% | 9 | 11.3% | 25 | 11.9% |  |
|  | 50.0 – 59.9 | 43 | 33.1% | 28 | 35.0% | 71 | 33.8% |  |
|  | 60.0 – 69.9  ≥ 70.0 | 47  24 | 36.1%  18.5% | 29  14 | 36.2%  17.5% | 76  38 | 36.2%  18.1% | .989 |
|  |  |  |  |  |  |  |  |  |
|  | Total | 130 | 100.0% | 80 | 100.0% | 210 | 100.0% |  |
| KPS at start SPC | < 70  70 – 80  90 – 100  No data available | 30  36  4  60 | 23.1%  27.7%  3.1%  46.1% | 36  28  6  10 | 45.0%  35.0%  7.5%  12.5% | 66  64  10  70 | 31.4%  30.5%  4.8%  33.3% | <.001 |
|  |  |  |  |  |  |  |  |  |
|  | Total | 130 | 100.0% | 80 | 100.0% | 210 | 100.0% |  |
| Start of inpatient palliative consultations before death in weeks | < 5  5 – 12  > 12 | 0  0  0 | 0.0%  0.0%  0.0% | 30  22  28 | 37.5%  27.5%  35.0% | 30  22  28 | 37.5%  27.5%  35.0% |  |
|  |  |  |  |  |  |  |  |  |
|  | Total | 0 | 0.0% | 80 | 100.0% | 80 | 100.0% |  |
| Number of inpatient  palliative consultations | 0 | 130 | 100.0% | 0 | 0.0% | 130 | 61.9% |  |
|  | 1 | 0 | 0.0% | 25 | 31.3% | 25 | 11.9% | <.001 |
|  | 2 | 0 | 0.0% | 22 | 27.5% | 22 | 10.5% |  |
|  | > 2 | 0 | 0.0% | 33 | 41.2% | 33 | 15.7% |  |
|  | Total | 130 | 100.0% | 80 | 100.0% | 210 | 100.0% |  |

**Abbreviation:** SPC, specialized palliative care; n, valid number; X^2^, Pearson’s Chi-square test; KPS, Karnofsky Performance Scale; SPC, specialized palliative care.

**Supplementary Table 10** Indications of inpatient palliative consultation (*n* = 80).

|  |  |  | Valid Number | Percent |
| --- | --- | --- | --- | --- |
| Pain |  | No | 60 | 75.0% |
|  |  | Yes | 20 | 25.0% |
| Neurological or psychiatric symptoms |  | No | 57 | 71.2% |
|  |  | Yes | 23 | 28.8% |
| Cardiac or respiratory symptoms |  | No | 78 | 97.5% |
|  |  | Yes | 2 | 2.5% |
| Gastrointestinal symptoms |  | No | 68 | 85.0% |
|  |  | Yes | 12 | 15.0% |
| Wounds |  | No | 80 | 100.0% |
|  |  | Yes | 0 | 0.0% |
| Urogenital symptoms |  | No | 80 | 100.0% |
|  |  | Yes | 0 | 0.0% |
| Other symptoms |  | No | 71 | 88.2% |
|  |  | Yes | 9 | 11.3% |
| Consultation at first diagnosis |  | No | 72 | 90.0% |
|  |  | Yes | 8 | 10.0% |
| Therapy goal setting |  | No | 54 | 67.5% |
|  |  | Yes | 26 | 32.5% |
| Care coordination |  | No | 3 | 3.8% |
|  |  | Yes | 77 | 96.2% |
| Other indications* |  | No | 23 | 28.8% |
|  |  | Yes | 57 | 71.2% |
|  |  | Total | 80 | 100.0% |

*e.g. Domestic support, psychosocial support of caregiver

**Supplementary Table 11** Characteristics of patients with stay at PCU (cohort IV, *n* = 210).

|  | | Inpatient palliative consultation | | | | | |  |
| --- | --- | --- | --- | --- | --- | --- | --- | --- |
|  |  | No | | Yes | | Total | | X^2^ |
|  |  | n | (%) | n | (%) | n | (%) | *p* |
| Sex | Male | 69 | 53.9% | 49 | 59.8% | 118 | 56.2% | .405 |
|  | Female | 59 | 46.1% | 33 | 40.2% | 92 | 43.8% |  |
|  | Total | 128 | 100.0% | 82 | 100.0% | 210 | 100.0% |  |
| Age at diagnosis in years | 20.0 – 49.9 | 12 | 9.4% | 13 | 15.9% | 25 | 11.9% |  |
|  | 50.0 – 59.9 | 44 | 34.4% | 27 | 32.9% | 71 | 33.8% |  |
|  | 60.0 – 69.9  ≥ 70.0 | 46  26 | 35.9%  20.3% | 30  12 | 36.6%  14.6% | 76  38 | 36.2%  18.1% | .442 |
|  |  |  |  |  |  |  |  |  |
|  |  | 128 | 100.0% | 82 | 100.0% | 210 | 100.0% |  |
| KPS at start SPC | < 70  70 – 80  90 – 100  No data available | 45  39  4  40 | 35.1%  30.5%  3.1%  31.3% | 21  25  6  30 | 25.6%  30.5%  7.3%  36.6% | 66  64  10  70 | 31.4%  30.5%  4.8%  33.3% | .293 |
|  |  |  |  |  |  |  |  |  |
|  | Total | 128 | 100.0% | 82 | 100.0% | 210 | 100.0% |  |
| Duration of stay in PCU | ≤ 7  8 – 14  > 14 | 0  0  0 | 0.0%  0.0%  0.0% | 29  37  16 | 35.4%  45.1%  19.5% | 29  37  16 | 35.4%  45.1%  19.5% | - |
|  |  |  |  |  |  |  |  |  |
|  | Total | 0 | 0.0% | 82 | 100.0% | 82 | 100.0% |  |

**Abbreviation:** PCU, palliative care unit; SPC, specialized palliative care; n, valid number; X^2^, Pearson’s Chi-square test; KPS, Karnofsky Performance Scale.

**Supplementary Table 12** Indications of PCU (*n* = 82).

|  |  |  | Valid Number | Percent |
| --- | --- | --- | --- | --- |
| Pain |  | No | 47 | 57.3% |
|  |  | Yes | 17 | 20.7% |
|  |  | No data available | 18 | 22.0% |
| Neurological or psychiatric symptoms |  | No | 39 | 47.5% |
|  |  | Yes | 25 | 30.5% |
|  |  | No data available | 18 | 22.0% |
| Cardiac or respiratory symptoms |  | No | 60 | 73.1% |
|  |  | Yes | 4 | 4.9% |
|  |  | No data available | 18 | 22.0% |
| Gastrointestinal symptoms |  | No | 57 | 69.5% |
|  |  | Yes | 7 | 8.5% |
|  |  | No data available | 18 | 22.0% |
| Wounds |  | No | 64 | 78.0% |
|  |  | Yes | 0 | 0.0% |
|  |  | No data available | 18 | 22.0% |
| Urogenital symptoms |  | No | 63 | 76.8% |
|  |  | Yes | 1 | 1.2% |
|  |  | No data available | 18 | 22.0% |
| Other symptoms |  | No | 10 | 12.2% |
|  |  | Yes | 54 | 65.8% |
|  |  | No data available | 18 | 22.0% |
|  |  | Total | 82 | 100.0% |

**Abbreviation:** PCU, palliative care unit.

**Supplementary Table 13** Characteristics of patients with SPOC (cohort IV, *n* = 210).

|  | | SPOC | | | | | |  |
| --- | --- | --- | --- | --- | --- | --- | --- | --- |
|  |  | No | | Yes | | Total | | X^2^ |
|  |  | n | (%) | n | (%) | n | (%) | *p* |
| Sex | Male | 39 | 54.2% | 79 | 57.2% | 118 | 56.2% | .669 |
|  | Female | 33 | 45.8% | 59 | 42.8% | 92 | 43.8% |  |
|  | Total | 72 | 100.0% | 138 | 100.0% | 210 | 100.0% |  |
| Age at diagnosis in years | 20.0 – 49.9 | 9 | 12.5% | 16 | 11.6% | 25 | 11.9% |  |
|  | 50.0 – 9.9 | 22 | 30.6% | 49 | 35.5% | 71 | 33.8% | .897 |
|  | 60.0 – 69.9  ≥ 70.0 | 28  13 | 38.8%  18.1% | 48  25 | 34.8%  18.1% | 76  38 | 36.2%  18.1% |  |
|  |  |  |  |  |  |  |  |  |
|  | Total | 72 | 100.0% | 138 | 100.0% | 210 | 100.0% |  |
| KPS at start SPC | < 70  70 – 80  90 – 100  No data available | 22  17  5  28 | 30.6%  23.6%  6.9%  38.9% | 44  47  5  42 | 31.9%  34.1%  3.6%  30.4% | 66  64  10  70 | 31.4%  30.5%  4.8%  33.3% | .280 |
|  |  |  |  |  |  |  |  |  |
|  | Total | 72 | 100.0% | 138 | 100.0% | 210 | 100.0% |  |
| Duration of SPOC in weeks | < 2  2 – 4  > 4 | 0  0  0 | 0.0%  0.0%  0.0% | 33  36  68 | 24.1%  26.3%  49.6% | 33  36  68 | 24.1%  26.3%  49.6% | - |
|  |  |  |  |  |  |  |  |  |
|  | Total | 0 | 0.0% | 137 | 100.0% | 137 | 100.0% |  |
| Number of total contacts with SPOC | < 10 | 0 | 0.0% | 38 | 27.7% | 38 | 27.7% | - |
|  | 10 – 30 | 0 | 0.0% | 46 | 33.6% | 46 | 33.6% |  |
|  | > 30 | 0 | 0.0% | 32 | 23.4% | 32 | 23.4% |  |
|  | No data available | 0 | 0.0% | 21 | 15.3% | 21 | 15.3% |  |
|  | Total | 0 | 0.0% | 137 | 100.0% | 137 | 100.0% |  |

**Abbreviation:** SPOC, specialized palliative outpatient care; SPC, specialized palliative care; n, valid number; X^2^, Pearson’s Chi-square test; KPS, Karnofsky Performance Scale.

**Supplementary Table 14** Indications of SPOC (*n* = 138).

|  |  |  | Valid Number | Percent |
| --- | --- | --- | --- | --- |
| Pain |  | No | 71 | 51.4% |
|  |  | Yes | 61 | 44.2% |
|  |  | No data available | 6 | 4.4% |
| Neurological or psychiatric symptoms |  | No | 24 | 17.4% |
|  |  | Yes | 108 | 78.3% |
|  |  | No data available | 6 | 4.3% |
| Cardiac or respiratory symptoms |  | No | 121 | 87.7% |
|  |  | Yes | 11 | 8.0% |
|  |  | No data available | 6 | 4.3% |
| Gastrointestinal symptoms |  | No | 99 | 71.7% |
|  |  | Yes | 33 | 23.9% |
|  |  | No data available | 6 | 4.5% |
| Wounds |  | No | 130 | 94.2% |
|  |  | Yes | 2 | 1.5% |
|  |  | No data available | 6 | 4.3% |
| Urogenital symptoms |  | No | 130 | 94.2% |
|  |  | Yes | 2 | 1.5% |
|  |  | No data available | 6 | 4.3% |
| Other symptoms |  | No | 72 | 52.2% |
|  |  | Yes | 60 | 43.5% |
|  |  | No data available | 6 | 4.3% |
|  |  | Total | 138 | 100.0% |

**Abbreviation:** SPOC, specialized palliative outpatient care.

**Supplementary Table 15** Characteristics of patients with hospice care (cohort IV, *n* = 210).

|  | | Hospice care | | | | | |  |
| --- | --- | --- | --- | --- | --- | --- | --- | --- |
|  |  | No | | Yes | | Total | | X^2^ |
|  |  | n | (%) | n | (%) | n | (%) | *p* |
| Sex | Male | 104 | 58.1% | 14 | 45.2% | 118 | 56.2% | .180 |
|  | Female | 75 | 41.9% | 17 | 54.8% | 92 | 43.8% |  |
|  | Total | 72 | 100.0% | 138 | 100.0% | 210 | 100.0% |  |
| Age at diagnosis in years | 20.0 – 49.9 | 21 | 11.7% | 4 | 12.9% | 25 | 11.9% |  |
|  | 50.0 – 59.9 | 61 | 34.1% | 10 | 32.3% | 71 | 33.8% | .818 |
|  | 60.0 – 69.9  ≥ 70.0 | 63  34 | 35.2%  19.0% | 13  4 | 41.9%  12.9% | 76  38 | 36.2%  18.1% |  |
|  |  |  |  |  |  |  |  |  |
|  | Total | 179 | 100.0% | 31 | 100.0% | 210 | 100.0% |  |
| KPS at start SPC | < 70  70 – 80  90 – 100  No data available | 57  52  8  62 | 31.8%  29.1%  4.5%  34.6% | 9  12  2  8 | 29.0%  38.7%  6.5%  25.8% | 66  64  10  70 | 31.4%  30.5%  4.8%  33.3% | .634 |
|  |  |  |  |  |  |  |  |  |
|  | Total | 179 | 100.0% | 31 | 100.0% | 210 | 100.0% |  |
| Duration of stay in hospice in weeks | ≤ 2  > 2 | 0  0 | 0.0%  0.0% | 14  17 | 45.2%  54.8% | 14  17 | 45.2%  54.8% | - |
|  | Total | 0 | 0.0% | 31 | 100.0% | 31 | 100.0% |  |
| **Abbreviation:** SPC, specialized palliative care; n, valid number; X^2^, Pearson’s Chi-square test; KPS, Karnofsky Performance Scale. | | | | | | | | |

**Supplementary Table 16** Description of tumor-specific therapy in the last 30 days prior to death in patients with SPC vs. without (cohort I, *n* = 274).

|  | | SPC | | | | | |  |
| --- | --- | --- | --- | --- | --- | --- | --- | --- |
|  |  | No | | Yes | | Total | | X^2^ |
|  |  | n | (%) | n | (%) | n | (%) | *p* |
| Tumor-specific therapy in the last 30 days prior to death | No | 40 | 62.5% | 175 | 83.3% | 215 | 78.5% | <0.001 |
|  | Yes | 24 | 37.5% | 35 | 16.7% | 59 | 21.5% |  |
|  | Total | 64 | 100.0% | 210 | 100.0% | 274 | 100.0% |  |
| **Abbreviation:** SPC, specialized palliative care; n, valid number; X^2^, Pearson’s Chi-square test; SPC, specialized palliative care. | | | | | | | | |

**Supplementary Table 17** Cox proportional-hazards regression model of the influence of clinical aspects on PFS (cohort I with *n* = 274).

| Variable | Category (*n*) | Univariable cox  regression analysis | | | | Multivariable cox  regression analysis | | | |  |
| --- | --- | --- | --- | --- | --- | --- | --- | --- | --- | --- |
|  |  | *p* | HR | Lower 95%-CI | Upper 95%-CI | *p* | HR | Lower 95%-CI | Upper 95%-CI |  |
| SPC | No (64) |  | 1.000 |  |  |  | 1.000 |  |  |  |
|  | Yes (210) | 0.776 | 0.960 | 0.724 | 1.272 | 0.496 | 0.906 | 0.683 | 1.203 |  |
| Sex | Male (158) |  | 1.000 |  |  |  | 1.000 |  |  |  |
|  | Female (116) | 0.200 | 0.854 | 0.670 | 1.087 | 0.246 | 0.864 | 0.674 | 1.106 |  |
| Age at first diagnosis^2^ |  | 1.198 | 1.007 | 0.996 | 1.018 | 0.010 | 1.014 | 1.003 | 1.026 |  |
| KPS^3^ at first diagnosis |  | 0.863 | 0.999 | 0.989 | 1.009 | 0.905 | 0.999 | 0.989 | 1.010 |  |
| *MGMT* promoter methylation | No (157) |  | 1.000 |  |  |  | 1.000 |  |  |  |
|  | Yes (117) | <0.001 | 0.440 | 0.339 | 0.571 | <0.001 | 0.401 | 0.306 | 0.527 |  |
| Macroscopic complete resection | No (157) |  | 1.000 |  |  |  | 1.000 |  |  |  |
|  | Yes (117) | 0.002 | 0.683 | 0.535 | 0.870 | <0.001 | 0.651 | 0.509 | 0.833 |  |

**Abbreviations:** PFS, progression-free survival; HR, Hazard Ratio; CI, Confidence Interval; KPS, Karnofsky Performance Scale; SPC, specialized palliative care.

^2^Age, as a continuous variable.

^3^KPS, as a categorical variable with 10 points increments.


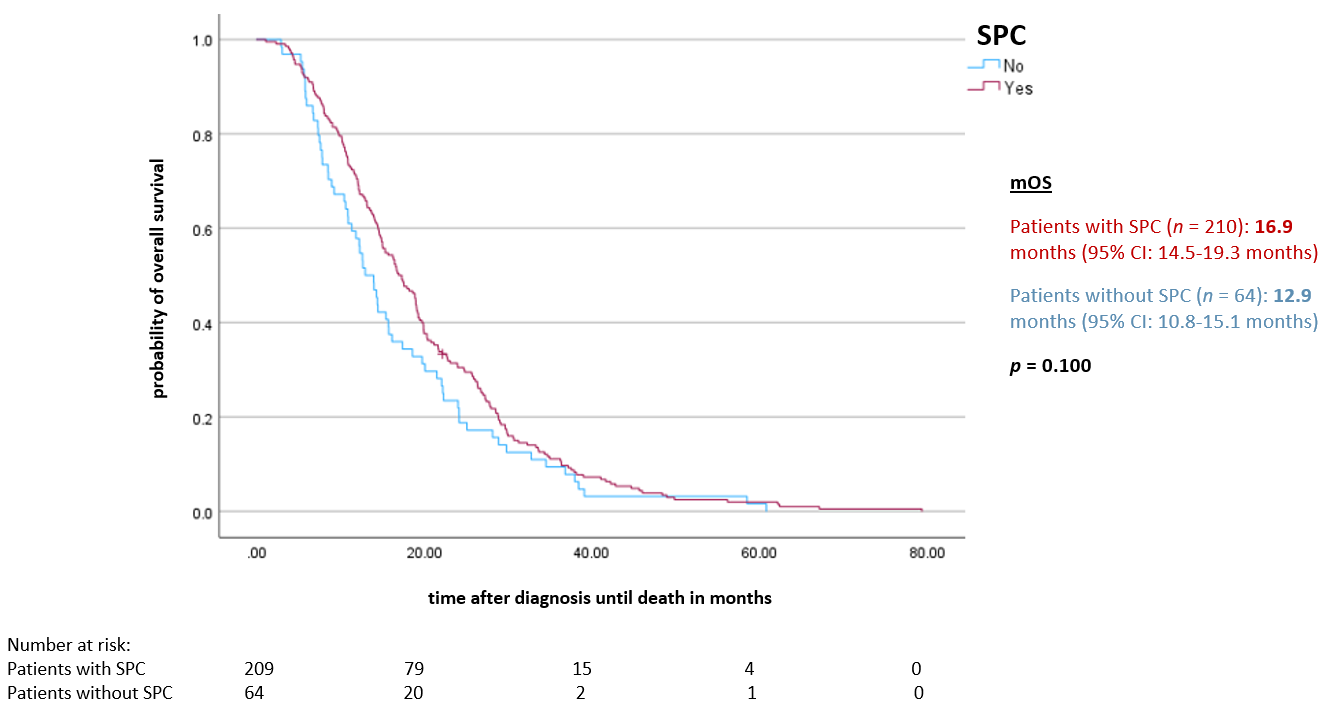
**Supplementary Figure 1** Kaplan-Meier estimation of OS for patients with SPC vs. without.

**Abbreviations:** OS, overall survival; SPC, specialized palliative care; mOS, median overall survival.


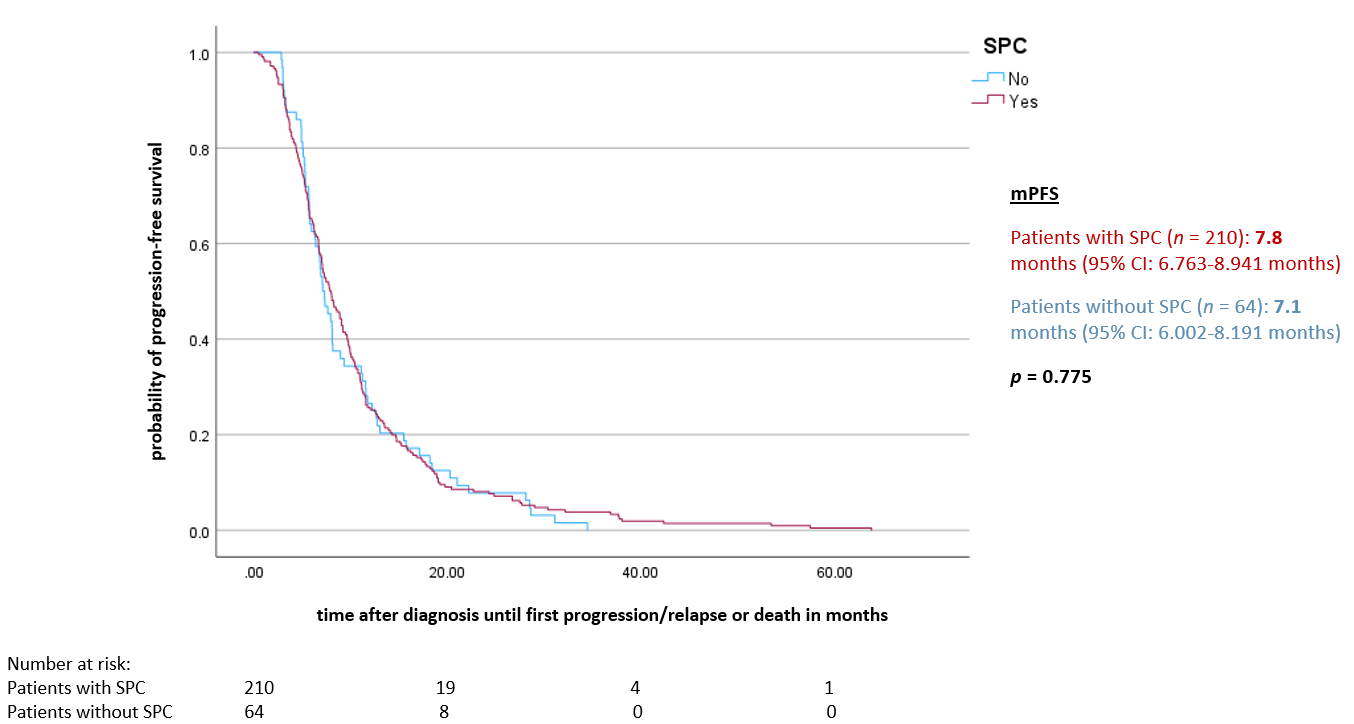
**Supplementary Figure 2** Kaplan-Meier estimation of PFS for patients with SPC vs. without. The multivariable Cox regression model revealed a longer PFS (HR 0.906; 95% CI: 0.683-1.203) for patients with SPC compared to patients without (*p* = 0.496).

**Abbreviations:** PFS, progression-free survival; SPC, specialized palliative care; mPFS, median progression-free survival.
